# Prokaryotic Capability to Use Organic Substrates Across the Global Tropical and Subtropical Ocean

**DOI:** 10.3389/fmicb.2020.00918

**Published:** 2020-06-04

**Authors:** Maria Montserrat Sala, Clara Ruiz-González, Encarna Borrull, Iñigo Azúa, Zuriñe Baña, Begoña Ayo, X. Antón Álvarez-Salgado, Josep M. Gasol, Carlos M. Duarte

**Affiliations:** ^1^Department of Marine Biology and Oceanography, Institut de Ciències del Mar, Consejo Superior de Investigaciones Científicas, Barcelona, Spain; ^2^Department of Immunology, Microbiology, and Parasitology, Faculty of Science and Technology, University of the Basque Country UPV/EHU, Leioa, Spain; ^3^Research Centre for Experimental Marine Biology and Biotechnology PiE-UPV/EHU, Plentzia, Spain; ^4^Departament of Oceanography, Instituto de Investigacións Mariñas (IIM-CSIC), Vigo, Spain; ^5^Centre for Marine Ecosystems Research, School of Science, Edith Cowan University, Joondalup, WA, Australia; ^6^Red Sea Research Center, King Abdullah University of Science and Technology, Thuwal, Saudi Arabia; ^7^Department of Global Change Research, Instituto Mediterráneo de Estudios Avanzados—Universitat de les Illes Balears, Consejo Superior de Investigaciones Científicas, Esporles, Spain

**Keywords:** bathypelagic, Biolog, functional profiles, global ocean, marine prokaryotic communities, acetic acid, bacterioplankton

## Abstract

Prokaryotes play a fundamental role in decomposing organic matter in the ocean, but little is known about how microbial metabolic capabilities vary at the global ocean scale and what are the drivers causing this variation. We aimed at obtaining the first global exploration of the functional capabilities of prokaryotes in the ocean, with emphasis on the under-sampled meso- and bathypelagic layers. We explored the potential utilization of 95 carbon sources with Biolog GN2 plates^®^ in 441 prokaryotic communities sampled from surface to bathypelagic waters (down to 4,000 m) at 111 stations distributed across the tropical and subtropical Atlantic, Indian, and Pacific oceans. The resulting metabolic profiles were compared with biological and physico-chemical properties such as fluorescent dissolved organic matter (DOM) or temperature. The relative use of the individual substrates was remarkably consistent across oceanic regions and layers, and only the Equatorial Pacific Ocean showed a different metabolic structure. When grouping substrates by categories, we observed some vertical variations, such as an increased relative utilization of polymers in bathypelagic layers or a higher relative use of P-compounds or amino acids in the surface ocean. The increased relative use of polymers with depth, together with the increases in humic DOM, suggest that deep ocean communities have the capability to process complex DOM. Overall, the main identified driver of the metabolic structure of ocean prokaryotic communities was temperature. Our results represent the first global depiction of the potential use of a variety of carbon sources by prokaryotic communities across the tropical and the subtropical ocean and show that acetic acid clearly emerges as one of the most widely potentially used carbon sources in the ocean.

## Introduction

Marine heterotrophic prokaryotes are responsible for most of the assimilation and the transformation of dissolved organic matter (DOM) in the ocean. Their activity facilitates the transformation from DOM to particulate organic matter (Azam et al., 1983) or the remineralization of DOM to its inorganic constituents (Ducklow et al., 1986). DOM is a large, complex pool, including thousands of compounds (Dittmar and Paeng, 2009; Arrieta et al., 2015) varying greatly in their reactivity and capability to support prokaryotic growth and metabolism. In the open ocean, the availability and the chemical structure of most DOM depends on biogeochemical processes such as phytoplankton production, grazing, viral lysis, and nutrient availability among other factors (Nagata, 2000). Its effective consumption by prokaryotes will depend on the metabolic potential of the community members as well as on the environmental variables that modulate prokaryotic activity and physiology. Despite the relevance of prokaryotic DOM utilization in controlling the nature and the fate of carbon in the global ocean, we still know very little about how the use of different carbon sources varies along environmental gradients or over large spatial scales.

Although the composition of marine prokaryotic communities has been explored in recent years at the global ocean scale (Pommier et al., 2007; Ladau et al., 2013; Sunagawa et al., 2015; Salazar et al., 2016; Mestre et al., 2018), linking taxonomy with community functionality has become an elusive goal. Whereas some studies reported changes in function to be associated to shifts in the prokaryotic assemblage taxonomic composition (Langenheder et al., 2005; Teske et al., 2011), others have shown largely uncoupled patterns at various spatial scales (Comte and del Giorgio, 2010; Lear et al., 2014; Ruiz-González et al., 2015). Since the uptake of various DOM components differs among various groups of marine prokaryotes (Martinez et al., 1996; Cottrell and Kirchman, 2000; Ruiz-González et al., 2012; Sarmento et al., 2013), it is not straightforward to infer the utilization of different carbon sources from community composition or even genomic composition.

The quality of DOM has been shown to affect prokaryotic growth (e.g., Sarmento et al., 2013). However, less is known on the influence of DOM quality on the metabolic capabilities of microbial communities beyond bulk community properties such as prokaryotic heterotrophic production or respiration. Fluorescent dissolved organic matter (FDOM) emerged as a main factor in shaping the spatial structuring of the functional capacities of hundreds of freshwater prokaryotic communities, whereas it did not explain the observed variation in taxonomic composition (Ruiz-González et al., 2015). Several studies have shown that both DOM bioavailability and their composition vary only slightly across different aquatic systems (Søndergaard et al., 1995; Zark and Dittmar, 2018) and between coastal and open ocean ecosystems (Seidel et al., 2015). In the global ocean, results of the Malaspina-2010 Expedition showed also weak differences in FDOM, often used as a proxy for some DOM compound classes, as well as in phytoplankton composition across the epipelagic ocean (Catalá et al., 2016; Estrada et al., 2016). In contrast, differences in FDOM between the epipelagic and the bathypelagic ocean during the Malaspina Expedition (Catalá et al., 2015, 2016) suggest that the patterns of substrate use may change with depth due to the vertical variations in the quality of DOM. Thus, the exploration of the factors that shape the biogeography of the metabolic potential of natural microbial communities may unveil relevant drivers that are not apparent from the study of their taxonomic composition or DOM quality. Moreover, a functional profiling of prokaryotic substrate use capability may help identify key substrates that can be universally used by prokaryotic communities.

Assessing the use of organic matter compounds by natural prokaryotic communities has proven to be challenging. Current approaches include the use of labeled (radioisotope or fluorogenic) substrates (e.g., Hoppe, 1983; Sala and Güde, 1999; Arnosti, 2011) or approaches using changes in DOM profiles, such as those resolved through Fourier-transform ion cyclotron resonance mass spectrometry during incubations (Dittmar et al., 2008). However, the use of labeled substrates is limited to a few, generally up to 10, compounds (e.g., Sala et al., 2010). Other approaches using DOM profiling remain semi-quantitative and also fail to resolve the low molecular weight compounds (Nebbioso and Piccolo, 2013), which can comprise a significant fraction of the DOM pool. Alternative avenues, using metagenomic or transcriptomic approaches (e.g., Rivers et al., 2013), are promising. However, they are complex, time-expensive, and limited by the fact that gene expression does not necessarily translate into substrate use (Rocca et al., 2015). In addition, the functional annotation of genes does not yet allow a comprehensive understanding of the potential substrate use and associated transformations.

Although originally designed to quickly identify prokaryotic isolates in clinical pathology (Armon et al., 1990), Biolog microplates^TM^ were soon applied for the rapid screening of organic substrate use in natural microbial communities including freshwater, soils, and the rhizosphere (Garland and Mills, 1991). The Biolog methods are based on microplates seeded with different putative organic substrates and an indicator that shifts absorbance when the substrate is used (i.e., a tetrazolium salt that is reduced to formazan). These microplates are commercially available with up to 95 different substrates and blanks and are affordable and easy to operate, thereby allowing for a high-throughput assessment of substrate use. The technique is a culture-dependent method that requires relatively long incubations to achieve sufficient biomass that could be detectable (Konopka et al., 1998). However, it has been shown to be useful to characterize differences in functional diversity among microbial communities through their capability to use a range of sole carbon sources (Garland and Mills, 1991; Preston-Mafham et al., 2002).

Most previous studies with Biolog microplates dealt with soil (Zak et al., 1994; Wunsche et al., 1995) or freshwater (Grover and Chrzanowski, 2000; Comte and del Giorgio, 2010; Lear et al., 2014) microbial communities, but their use to profile the metabolic potential of marine prokaryotic communities remains poorly explored (Supplementary Table S1). Specifically, microplate-based studies on the substrate use capability of marine prokaryotic pelagic communities have been restricted to the epipelagic layer of coastal waters (Sala et al., 2005a, 2006; They et al., 2013; Boras et al., 2015), polar marine ecosystems (Tam et al., 2003; Sala et al., 2005b, 2008, 2010; Fernández-Gómez et al., 2014), and the microlayer of coastal and offshore ecosystems (Wurl et al., 2016). So far, there is no study exploring the variations of the prokaryotic metabolic profiles of communities at the global ocean scale and specifically in the deep ocean.

We here characterize the global patterns of organic substrate utilization by pelagic prokaryotes across 441 communities sampled from the surface to the bathypelagic waters of the Atlantic, Indian, and Pacific tropical or subtropical oceans. Water samples were collected during the Malaspina-2010 Circumnavigation Expedition (Duarte, 2015), and the metabolic profiles of the studied communities were determined by their capability to use a range of sole carbon sources based on Biolog GN2 microplates. The global spatial and vertical variations in these profiles were related to changes in geographical or environmental variables to provide the first comprehensive metabolic characterization of the tropical and the subtropical oceanic microbiome at the global scale. We hypothesize that, based mainly on the patterns of FDOM and prokaryotic community composition during the Malaspina-2010 cruise (Catalá et al., 2016; Salazar et al., 2016; Ruiz-González et al., 2019), the use of different carbon substrates will vary weakly among oceans while showing pronounced changes with depth.

## Materials and Methods

### Sampling

**FIGURE 1:**
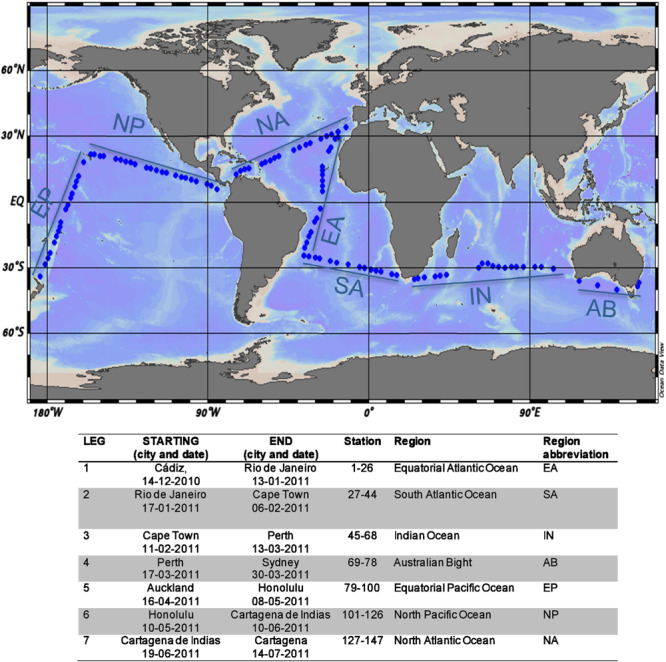
Map showing the sampling stations across the tropical and the subtropical ocean, indicating the different oceanic regions (Ocean Data View; Schlitzer, 2013). Dates and start and end locations, stations, and regions covered during each leg are provided in the table below. Region delimitation as in Pernice et al. (2015).

A total of 441 water samples were collected during the Malaspina-2010 Expedition to analyze the microbial utilization of carbon sources. The samples were obtained from 111 different stations distributed across the tropical and the subtropical oceans (Figure 1). Each station was sampled between 3 and 4,000 m, with a higher sampling intensity in the upper 1,000 m. The number of depths sampled per station ranged from one to eight. Surface (3 m) samples were collected with a 30-L Niskin bottle, and deeper samples were collected using a rosette fitted with 24 12-L Niskin bottles operated from a Seabird Rosette sampling system. Temperature and salinity were measured with a calibrated SeaBird 9/11-plus CTD. Samples for quantification of chlorophyll *a* and DOC concentration, FDOM intensity, prokaryotic and viral abundances, and prokaryotic heterotrophic production were also collected from each sampled depth (see Supplementary Methods). The mean values of the main parameters in the different layers [surface (3 m), the deep chlorophyll maximum (DCM, which ranged from 19 to 150 m), mesopelagic (sampled between 270 and 980 m), and bathypelagic (1,000–4,000 m)] are presented in Supplementary Table S2.

### Functional Profiles

The functional diversity of the studied prokaryotic assemblages was assessed with GN2 Biolog plates (Preston-Mafham et al., 2002). The choice of GN2 plates was based on the higher number of carbon sources present in the microplate (95) as compared to the most commonly used ECOplates. This allowed the exploration of a higher diversity of organic carbon in the previously unexplored oceans and especially in the bathypelagic layers.

Each of the 96 wells of a Biolog GN2 microplate contains a carbon source together with tetrazolium violet to indicate substrate oxidation. The list of substrates and categories is provided in Supplementary Table S3. After addition of 150 μl of sample in each well, the plates were incubated in the dark for 6 to 34 days (median incubation time, 20 days) at *in situ* temperature, and absorbance was regularly measured at 590-nm wavelength with a spectrophotometric microplate reader (ELX800 BIOTEK Instruments, Inc., Winooski, VT, United States). When a plateau of total absorbance of the plate (sum of all absorbances) was achieved, such measurement was retained for statistical analysis. The absorbance of the blanks (wells containing only water) was subtracted from the absorbance of each well, i.e., from each substrate. The absorbance of each substrate was standardized to the total absorbance of the plate and the data were reported as a percent of total absorbance of the plate (to avoid the effects of the different concentrations of prokaryotes in the inoculum, i.e., % absorbance of total color of the plate, and is referred to as relative use of each substrate hereafter). Further methodological details can be found in Sala et al. (2005a). The differences in incubation time due to differences in the time required to reach the maximum absorbance did not affect the results (Supplementary Figure S1). The prevalence of substrate use was defined as the number of samples in which the use of that substrate was detected (i.e., absorbance higher than the control).

For some analyses, such as the exploration of substrate utilization patterns by depth and ocean or in relation to environmental parameters, the substrates were grouped into functional categories, i.e., amino acids, carbohydrates, carboxylic acids, amines/amides, polymers, P-compounds, and miscellaneous (Supplementary Table S3). However, for the sake of a better representation of the results of the depth profiles, carbohydrates and carboxylic acids were further subdivided into two categories according to their high use (HU), which includes the 10 most used substrates within these categories, or low use (LU), which includes the rest of the substrates (Supplementary Table S4).

The diversity of single-source substrate use, which provides a metric of functional diversity for the communities tested, was calculated through the Shannon diversity index, *H*, as:

$$H=\sum_{i=1}^{S}p_{i}\times \ln p_{i}$$

where *p*_*i*_ is the fraction of the total absorbance contributed by a carbon source *i*, and the summation compiles the product of *p*_*i*_ and ln *p*_*i*_ for all 95 organic substrates tested.

### Statistical Analyses

The functional structure of all the studied communities was summarized by means of a principal component analysis (PCA) based on the correlation matrix of the percentage use of the 95 substrates. The *envfit* function (Vegan R package) was used to fit onto the ordination space the environmental predictors that best explained the distribution of the communities.

To avoid giving higher weight to the depths sampled with higher resolution in the vertical profiles, the depth patterns of relative use of each substrate in the upper 1,000 m were described by fitting a spline through the data. The fitted data were then averaged across substrates to characterize the depth patterns for categories of substrates. Splines were also used to describe the relation between temperature and substrates used after grouping the samples within 1°C temperature bins.

All statistical analyses were performed using the software JMP v.13 (SAS Institute), R 3.0.0. (R Core Team, 2017), and Statistica v. 8.0.

## Results and Discussion

### Widespread Use of Carbon Sources in the Ocean

On average, each of the 441 studied communities used 72% of the 95 substrates included in the Biolog microplates (median 74, range 5–95%), similar to the range reported for other environments such as soils and other ecosystems using the same approach [see Preston-Mafham et al. (2002) for a review]. All substrates were used by at least 41% (i.e., 181 communities) of the communities tested (*N* = 441 communities). No significant difference in the number of substrates used nor in the Shannon diversity index of single substrate use (average 3.44 ± 0.12) was found with depth, across ocean regions, or along the measured environmental gradients (ANOVA, *P* > 0.05).

**FIGURE 2:**
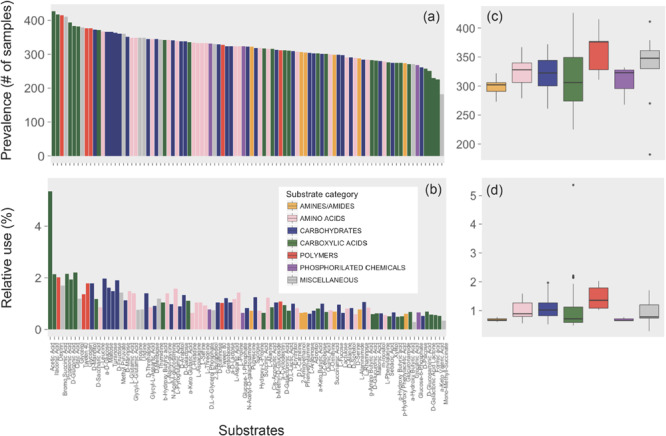
Prevalence (number of samples in which a given substrate was used) and mean relative use (absorbance of each substrate relative to the total absorbance of the sample) of the 95 substrates individually **(a,b)** or grouped by category **(c,d)**. The colors indicate different categories (see Supplementary Table S3). Boxplots represent median, first, and third quartiles and 1.5 × interquartile range (whiskers). Points beyond the end of the whiskers are outliers.

In general, the patterns of prevalence of the different substrate categories (see Supplementary Table S3 for a list of substrates and categories) across communities paralleled those of relative use, with polymers showing the highest prevalence and relative use and amines and amides showing the lowest prevalence and relative use across the communities tested (Figure 2). Beyond polymers, the highest relative use was found for carbohydrates, amino acids, and carboxylic acids, which are essential components of the organic matter pool in the ocean as inferred from the predicted substrate affinities of expressed transporter proteins (Bergauer et al., 2018).

### Acetate, the Main Carbon Source Used

Remarkably, all communities shared the utilization of one specific carbon source, acetic acid, which accounted for a relative use of, on average (±SE), 5.47 ± 0.27 across communities (i.e., mean of the percentage absorbance contribution to the total absorbance of the plate). This is at least twofold higher than that of the second most used carbon source (Figure 2b).

Although acetate is considered a fermentation product, rapid acetate uptake has been reported in several aquatic ecosystems, including surface oxic marine waters (Ho et al., 2002). Zhuang et al. (2019) recently revealed the high relevance of acetate as a metabolic substrate in the water column of the Gulf of Mexico. Acetate is primarily used as an energy source (evidenced by its high oxidation rates) and shows significant levels of assimilation into biomass, illustrating the potential significance of acetate as a carbon source that supports microbial growth (Zhuang et al., 2019). Indeed acetate addition has been proven to stimulate the growth of marine prokaryotes in enrichment experiments (Gómez-Consarnau et al., 2012). Several mechanisms of potential acetate production have been proposed for the ocean, such as DOC decomposition by photolysis (Mopper et al., 1991), release of algal intracellular pools of acetate by sloppy feeding, or deposition of dry aerosols and precipitation, which are rich in formic and acetic acid (Morikami et al., 1993). These processes are active in the surface ocean, but the high relative use of acetic acid has been found also in deep layers. Bathypelagic communities have been shown to be highly versatile metabolically and able to persist inactive or dormant for long periods during starvation (Sebastián et al., 2018), being able to quickly react to any source of labile or fresh carbon (Sebastián et al., 2019). Therefore, it is not unexpected that some members within the studied bathypelagic microbial communities can quickly use a labile carbon substrate such as acetic acid. In addition, we recently demonstrated that most of the bathypelagic prokaryotic taxa are also detected in surface waters and seem to be transported to deeper waters by being attached to sinking particles (Mestre et al., 2018). Consequently, there are certainly deep prokaryotic taxa that carry the potential to use carbon sources that derive from surface processes.

The ubiquity of potential acetic acid utilization found here (Figure 2a) agrees with our own data obtained in previous oceanographic cruises or experiments in which Biolog-GN2 plates were used. These included communities sampled in the Antarctic Ocean and the Mediterranean Sea as well as several experiments performed in the North Atlantic Ocean (Supplementary Figure S2), supporting that acetic acid is clearly the carbon source with the highest use potential. The comparison with data from other studies using Biolog plates is difficult since acetic acid is only present in GN and GN2 plates, which are no longer commercially available, but it is absent in the most commonly used Ecoplates (Supplementary Table S1). In addition, the only two published marine studies using GN and GN2 plates (Jellett et al., 1996; Wear et al., 2014) did not provide details on the utilization of the individual carbon sources (Supplementary Table S1).

The widespread use of acetic acid agrees with the fact that carboxylic acids are used by a wide variety of bacterial groups, from Flavobacteria to Alphaproteobacteria and even cyanobacteria [see Kujawinski (2011) for a review], all of which are ubiquitous members of marine bacterioplankton. Also, groups like the Alphaproteobacteria SAR11 or Roseobacter contain the genes for ABC transporters of small carboxylic acids such as acetate (Kujawinski, 2011).

### Spatial Variability in the Relative Use of Substrates Among Oceanic Regions

**FIGURE 3:**
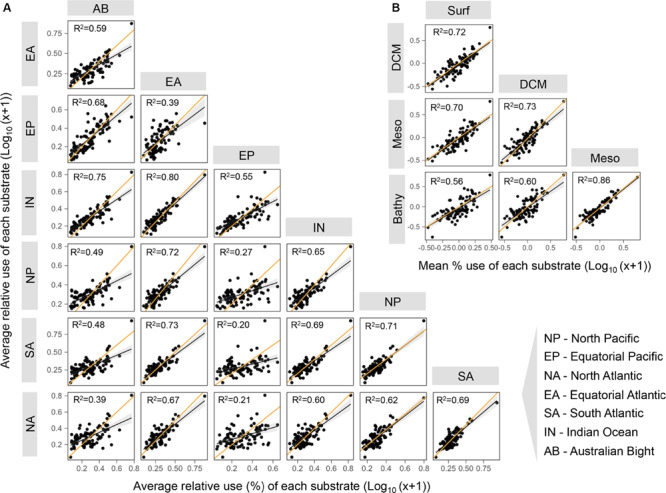
Pairwise comparisons between the relative use of each substrate averaged by oceanic region **(A)** or ocean layer **(B)**. The orange line indicates the 1:1 line. The black lines are linear regressions, significant in all cases (*p* < 0.0001). Layer abbreviations: Surf, surface; DCM, deep chlorophyll maximum; Meso, mesopelagic; Bathy, bathypelagic. The regions are as described in Figure 1.

**FIGURE 4:**
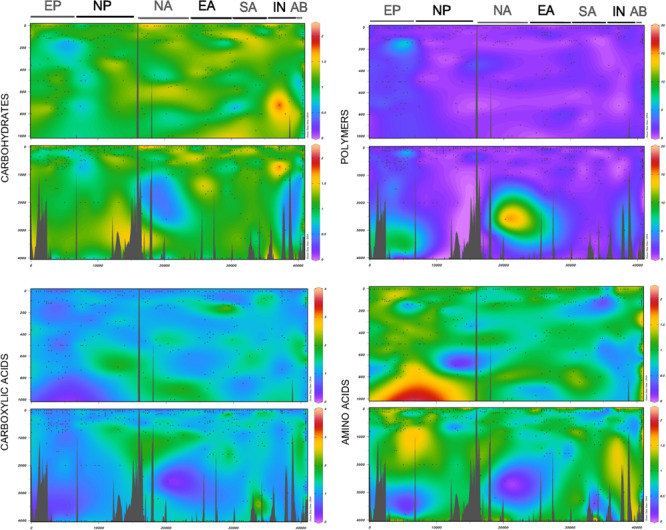
Relative use of different substrate categories along the entire expedition as visualized with Ocean Data View (Schlitzer, 2013). The different oceanic regions (see Figure 1 for descriptions) are indicated at the top of each graph and are ordered as in the map (Figure 1). Small dots indicate the sampling points. The upper panels show the upper part of the profile until 1,000 m, where higher-resolution profiles were performed, and the lower panels represent the entire profile from the surface until 4,000 m.

The patterns of use of the different substrates were generally similar across oceanic regions since we observed a good linear relationship between the mean relative use of the different regions for most of the 95 substrates (Figure 3A). Likewise, the relative use of the different substrate categories varied little among oceanic regions and with depth, with the exception of a few sites where particularly high or low substrate use was recorded (Figure 4). This lack of major regional differences in relative substrate use is consistent with the weak differences observed in the composition of FDOM (Catalá et al., 2016) and that of the phytoplankton assemblages (Estrada et al., 2016) during the Malaspina Expedition in epipelagic waters. With the exception of some particular stations, bacterioplankton composition was also rather constant across stations in surface waters (Ruiz-González et al., 2019) and in the bathypelagic layers (Salazar et al., 2016), supporting the relatively uniform pattern of functional diversity observed in our dataset.

The weakest relationships between the relative use of the different substrates from different regions were found when the Equatorial Pacific was compared to the rest of the regions (Equatorial Pacific vs. Equatorial Atlantic *R*^2^ = 0.39, vs. North Pacific *R*^2^ = 0.27, vs. South Atlantic *R*^2^ = 0.20, and vs. North Atlantic *R*^2^ = 0.21) (Figure 3A). Regarding the comparison of mean relative substrate use between ocean vertical layers (Figure 3B), we also observed strong significant relationships, suggesting a general consistency in the mean relative use of each substrate at the vertical scale. The lowest relationship was found between the surface and the bathypelagic (*R*^2^ = 0.56, Figure 3B), so we examined in detail the carbon sources that were significantly differently used in both layers (Supplementary Figure S3). We observed a significantly higher relative use of polymers in the Equatorial Pacific (in the surface and especially in the bathypelagic layers) than in other regions, with the highest ratio polymer/carbohydrate use both in the surface and in the bathypelagic layers (Supplementary Figure S3). In contrast, acetic acid showed a clearly lower relative use in the Equatorial Pacific than in other regions. Previous studies from the same expedition showed that, although the bathypelagic microbial communities were generally quite homogeneous, those in the Pacific Ocean differed (Salazar et al., 2016) and had the highest viral concentration and activity (Lara et al., 2017), high protistan abundance (Pernice et al., 2015), and a distinct bulk activity profile. Similarly, bacterial communities inhabiting the surface Equatorial Pacific showed a different structure, characterized by high abundances of specific taxa belonging to Oceanospirillales, Sphingomonadales, and picocyanobacteria that were rare across all the other open ocean stations studied (Ruiz-González et al., 2019). The control of the prokaryotic communities in the Equatorial Pacific by temperature and bottom-up sources was the lowest across the oceans sampled (Morán et al., 2017). Our evidence that the Equatorial Pacific Ocean harbors microbial communities that differ in metabolic profiles from those found elsewhere in the subtropical and the tropical ocean agrees with previous results from the same expedition which showed the existence of specific microbial communities in this region (Pernice et al., 2015; Lara et al., 2017; Ruiz-González et al., 2019).

### Variability in Relative Substrate Use With Depth

**FIGURE 5:**
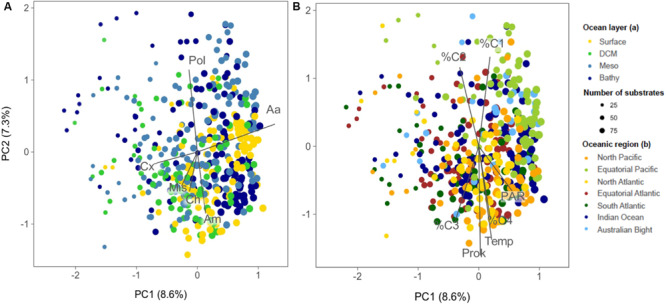
Principal component analysis (PCA) ordination of samples based on the functional structure of prokaryotic communities. The samples are labeled according to oceanic layer **(A)** or region **(B)**. The dot size is proportional to the number of substrates used per community. The vectors indicate the main substrate categories in **(A)** and the environmental variables that significantly fitted the ordination space in **(B)** (using the R *envfit* function). The size of the vector is proportional to the strength of the linear relationship of each variable. The first two PCA axes explain 16% of the variance. Pol, polymers; Aa, amino acids; Cx, carboxylic acids; Ch, carbohydrates; Am, amides/amines; Mis, miscellaneous;%C1, %C2, %C3, and %C4, percentage contribution of the fluorescent parallel factor analysis components C1, C2, C3, and C4 to the total DOM fluorescence; Temp, temperature; Prok, prokaryotic abundance; PAR, photosynthetically active radiation.

**FIGURE 6:**
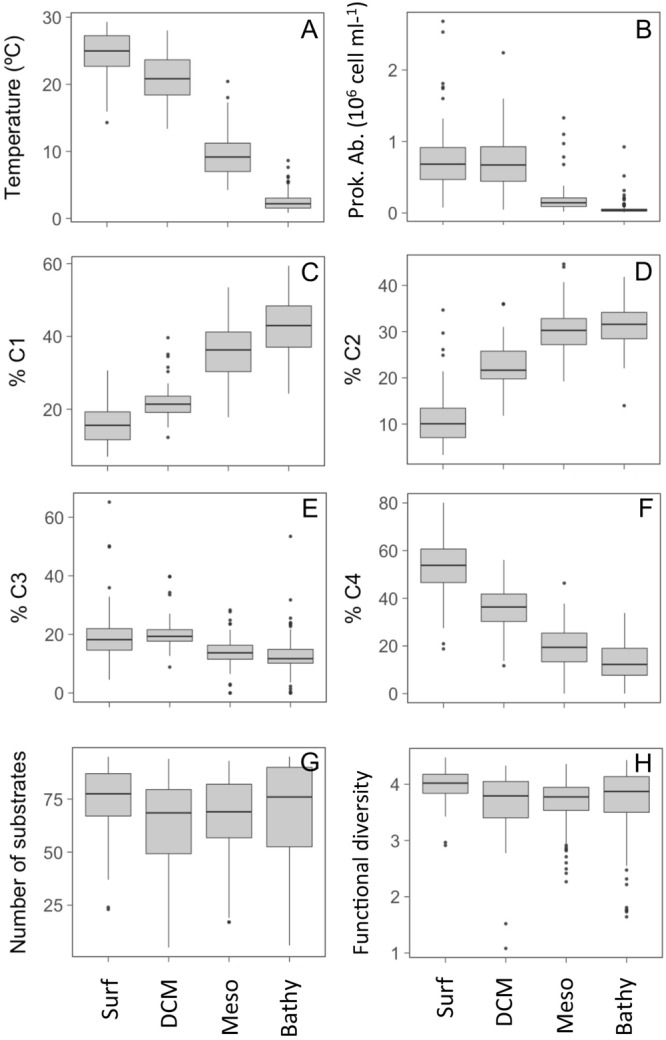
Variation across the four ocean layers (Surf, surface; DCM, deep chlorophyll maximum; Meso: mesopelagic; Bathy: bathypelagic) in **(A)** temperature, **(B)** prokaryotic abundance, **(C–F)** percentage contribution of the PARAFAC FDOM components C1, C2, C3, and C4 to the total DOM fluorescence, **(G)** number of Biolog substrates used, and **(H)** functional diversity (Shannon index) of each community.

The PCA showed no clear clustering of communities depending on the ocean layer considered (Figure 5A) and there were no clear vertical variations in the number of substrates used per community or in functional diversity (Figures 6G,H). This contrasts with the pronounced decreases in prokaryotic abundance with depth (Figures 6A,B) and, e.g. Aristegui (2009) and the increases in bacterial taxonomic and functional richness toward bathypelagic (Pommier et al., 2010) or mesopelagic (Sunagawa et al., 2015) waters as reported in previous studies. During the Malaspina Expedition, the prokaryotic communities were characterized in the surface (Ruiz-González et al., 2019) and the bathypelagic waters (Salazar et al., 2016), but a vertical characterization of the extent of the present study is only available from 8 stations (Mestre et al., 2018). As found by others, this vertical characterization of assemblages indicated that surface and DCM communities clustered together and differentiated from the meso- and bathypelagic assemblages. Such a clear vertical structuring of microbial communities and the lack of such obvious vertical variations in the overall metabolic profiles point to a high functional redundancy of the prokaryotic assemblages studied and suggest that, despite the vertical succession of taxa, most communities are able to potentially use the same organic substrates, at least those tested by the method used. Interestingly, this agrees with a metaproteomics study in the Atlantic Ocean which showed that the microbial communities from 100 to 5,000 m had similar transporter proteins (i.e., used similar substrates) throughout the water column despite large changes in community structure (Bergauer et al., 2018).

**FIGURE 7:**
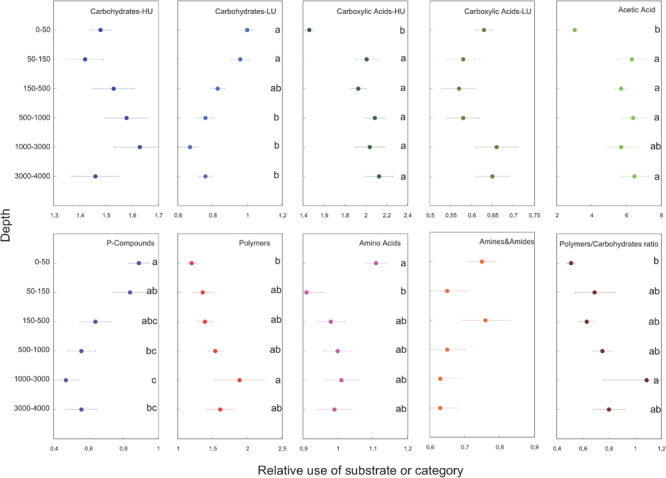
Variations in the relative use of the substrates within each category throughout the water column. The bars show the standard errors. The letters refer to the results of a *post hoc* Tukey’s test (*P* < 0.05). Different letters indicate significant differences among the considered depths. HU, high use; LU, low use (see Supplementary Table S4 for details).

When the 95 substrates were grouped by categories, we observed contrasting patterns of relative use with depth. In general, the relative use of some categories decreased or increased significantly from the communities sampled in the surface to the bathypelagic waters (Figure 7). A significantly higher relative use in the upper layers (50 m) was observed for P-compounds, amino acids, or amines/amides and also for LU-carbohydrates in the upper 150 m (Figure 7). Their higher relative use in the photic layer is consistent with reports showing that those categories contain labile compounds freshly produced by phytoplankton and that are rapidly used by prokaryotes (Nebbioso and Piccolo, 2013). In contrast, a lower relative use in the upper 50 m was found for HU-carboxylic acids (specifically for acetic acid) and also for polymers. The polymers showed an increased utilization with depth, and the ratio of polymers/carbohydrates use also increased with depth (ANOVA, *p* < 0.01) mainly due to the increase in polymer relative use (Figure 7). This pattern agrees with the reported increase in the abundance of transporters targeting aromatic compounds from surface to bathypelagic microbial communities sampled in the Atlantic Ocean (Bergauer et al., 2018).

A detailed analysis of the relative use of the individual carbon sources (Supplementary Figure S4) shows that the surface, and sometimes also the DCM, was characterized by a significantly higher relative use of carbohydrates (eight out of 28 substrates) such as *N*-acetyl-D-glucosamine, gentiobiose, D-lactose, and D-mellibiose or of P-compounds (two out of three substrates), α-D-glucose-6-phosphate or α-D-glucose-1-phosphate. In contrast, the bathypelagic prokaryotic communities had the potential to use a significantly higher number of carboxylic acids (seven out of 24) or polymers (two out of five), α-cyclodextrin and glycogen, than the upper layer communities. The high utilization of carbohydrates in the surface could be explained by the composition of marine dissolved organic matter in the epipelagic zone, which is dominated by the decay products of phytoplankton and consists of up to 40% carbohydrates, 25–50% proteins, and 5–25% lipids [Nebbioso and Piccolo (2013) and references therein].

Amino acids generally showed a higher relative use in surface and DCM waters compared to deeper layers, which agrees with the observed decrease in fluorescence intensity of the protein-like FDOM component (C4) from surface to bathypelagic waters (Figure 6F). Components C3 and C4 are considered to represent microbially produced fresh labile material, so their pattern of decreasing intensity suggests the production by phytoplankton communities in sunlit waters; conversely, components C1 and C2 represent more recalcitrant humic-like material (Catalá et al., 2016) and increased pronouncedly with depth (Figures 6C,D).

Several D-amino acids have shown decreasing concentrations with depth in the ocean (Kaiser and Benner, 2008; Kim et al., 2017). However, the D-amino acids (D-alanine and D-serine) were preferentially used in the mesopelagic and the bathypelagic layers (Supplementary Figure S4). Bacteria (Azúa et al., 2014) and Archaea, the latter abundant in bathypelagic waters, can consume D-amino acids (Teira et al., 2006), and the enantioselective utilization of D-amino acids was recently reported for bacteria isolated from deep-sea sediments (Kubota et al., 2016). Altogether this supports the preferential utilization of D-amino acids in deep waters as shown by our results.

The patterns of relative use of the different substrate categories were explored in detail within the upper 1,000 m by means of smoothed (i.e., average of spline fits) relative use data (Supplementary Figure S5). The smoothed data revealed the presence of peaks of relative substrate use below the DCM (which was located between 19 and 150 m across stations). At the base of the thermocline, around 200 m, the relative use of amines and amides showed a maximum and that of amino acids showed a minimum. This suggests that freshly produced amino acids in the photic layer are rapidly depleted and that communities shift toward the utilization of amines and amides (Supplementary Figure S5). This pattern is not apparent is not apparent from the vertical profiles of total dissolved organic nitrogen which results from the combined distribution of different compounds (Aluwihare et al., 2005).

Other peaks in potential utilization within the upper 1,000 m were observed at the deep scattering layer (DSL), which was located around 600 m (range 270 to 800 m). The DSL is an acoustic signature found across all oceans and formed by fish and zooplankton and is characterized by high biomass and daily vertical migrations (Aksnes et al., 2017). Migrating animals in the DSL may represent an overlooked source of carbon due to DOM release through excretion, fecal pellet dissolution, and/or mucus production (Calleja et al., 2018). The backscatter estimates measured with an echosounder are a proxy for the concentration of particles in the DSL layers. These values showed a positive correlation with prokaryotic abundance (*p* < 0.001), the humic-like C2 component of FDOM (*p* = 0.018), and the substrates D-serine (*p* = 0.010), gamma-hydroxybutyric acid (*p* = 0.012), and *N*-acetyl-D-galactosamine (*p* = 0.043). The use of polymers, HU-carbohydrates, and HU-carboxylic acids was maximal within the DSL (Supplementary Figure S5). This suggests an enrichment of these carbon sources in the DSL, which might be due to the increased organic matter concentration generated by trophic interactions in the layer and/or by active DOM release by migrating fish and zooplankton.

We acknowledge that these vertical patterns might be influenced by the effects of changing pressure, particularly in the case of deep-sea samples, as incubations were not performed at *in situ* pressure conditions. Current research seems to point toward a reduction of *in situ* activity of deep-sea bacteria under atmospheric conditions (Tamburini et al., 2013). Since our data are expressed relative to the total substrate use in each community, a reduction in total activity should not influence the relative use of the different substrates shown by our results. However, we cannot discard that exposure of bathypelagic communities to low pressures may have differentially affected specific taxa and their metabolism.

### Environmental Drivers of Microbial Functional Structure

As reported above, the principal component analysis performed based on the use of the 95 individual substrates did not show any clear clustering of communities with depth or oceanic region, and axes PC1 and PC2 accounted for a very low amount of total variance in the data, 8.6% for PC1 and 7.3% for PC2 (Figure 5). Prokaryotic abundance, temperature, PAR, and the percentage contribution of the four fluorescent components of DOM (C1, C2, C3, and C4), proxies of organic matter quality, were the variables that appeared to be significantly correlated to the ordination (Figure 5). Interestingly, the components C3 and C4 showed in the PCA opposite patterns to those of C2 and C1, although they were only weakly related to the ordination (envfit analysis *R*^2^ values = 0.05–0.07, all *p* < 0.01). As stated above, the proportion of C1 and C2, related to humic substances, increased toward the bathypelagic waters while that of C4, related to protein-like and labile compounds, showed maximal concentrations at the surface layers (Figures 6C–F). This clear vertical pattern in the quality of organic matter was not reflected in the changes in the number of substrates used nor in the Shannon functional diversity (Figures 6G,H). Only the higher relative use of amino acids in the surface (Figure 7) coincided with the higher proportion of protein-like FDOM compounds.

**FIGURE 8:**
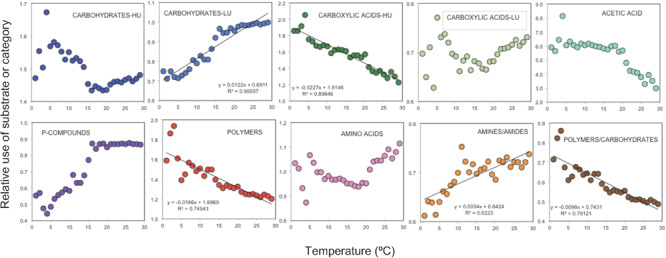
Relationships between the mean depth-smoothed relative use of the different substrate categories and temperature. Data from all depths and stations were pooled and averaged by 1°C temperature bins.

Temperature was the environmental variable that showed the strongest relationship with the PCA ordination, although it was also weak (envfit analysis *R*^2^ = 0.08, *p* < 0.001, Figure 5B). However, when grouping the substrates, the utilization patterns of some categories were clearly related to temperature (Figure 8). Using data averaged over 1°C bins and pooling all samples together, a positive relationship with temperature was found for the relative utilization of LU-carbohydrates or amines/amides (Figure 8). Conversely, the use of polymers, HU-carboxylic acids, and the ratio polymers/carbohydrates decreased with temperature (Figure 8). In general, we observed that the relationship between substrate use and temperature was not lineal during the whole temperature range but varied, showing different responses in cool waters (0–5°C), temperate waters (15–20°C), or warmer waters (20–30°C). For example, the utilization of P-compounds increased between 5 and 20°C, beyond which it did not change. The relative use of HU-carbohydrates seemed to increase up to 5°C, decreased pronouncedly with temperature between 5 and 20°C, and increased again in the warmest waters. Between 20 and 30°C, increases in temperature decreased the relative use of acetic acid and enhanced that of amino acids, while those of LU-carbohydrates or P-compounds were not affected (Figure 8). This complex control of substrate use by temperature may either be due to a direct and differential effect of temperature on metabolic processes (White et al., 1991; Pomeroy and Wiebe, 2001; Morán et al., 2017) or an indirect effect mediated by, e.g., changes in bacterial community structure along temperature gradients (e.g., Sunagawa et al., 2015; Ruiz-González et al., 2019). For example, in the epipelagic global ocean, an increase of microbial species richness was reported until 12°C, after which a negative correlation was found (Sunagawa et al., 2015). Finally, we acknowledge that other unmeasured environmental or biotic variables covarying with temperature may also have played a role.

## Conclusion

In summary, our global analysis of the metabolic capability of ocean prokaryotes reveals a widespread use of most of the studied carbon substrates by microbial communities across the subtropical and the tropical ocean. Among the substrates, acetic acid emerged as the most widely utilized compound across all sites and depths, with the exception of the Equatorial Pacific. Contrasting patterns of relative use with depth and temperature were found among major substrate categories, but there was little variability across ocean basins. The high-throughput assessment of the potential use of substrates with Biolog^®^ microplates, with its inherent limitations, has helped deliver a global functional profile of microbial communities across the global ocean that would have been difficult to obtain using other approaches.

## Data Availability Statement

The datasets generated for this study are available on request to the corresponding author.

## Author Contributions

MS, JG, and CD designed the work. MS, EB, IA, ZB, and BA performed the laboratory analysis. MS, CR-G, EB, and XÁ-S processed the data. MS wrote the manuscript with the help and the inputs of all co-authors.

## Conflict of Interest

The authors declare that the research was conducted in the absence of any commercial or financial relationships that could be construed as a potential conflict of interest.

## Supplementary Material

FIGURE S1 Relationship between the incubation time of the plates and the number of substrates used **(A)** and the functional diversity, Shannon **(B)**.

FIGURE S2 Correlation between the mean relative use of substrates during the Malaspina cruise vs. samples taken in the epipelagic waters in a cruise in the Antarctic and the sub-Antarctic ocean **(A)**, experiments with bathypelagic waters of the North Atlantic Ocean **(B)**, and in a cruise in the Mediterranean Sea **(C)**. The black arrows indicate acetic acid.

FIGURE S3 Mean relative use for each substrate in each oceanic region. The intensity of color in the boxes indicates the range of values, as indicated in the box. The letters refer to results of a *post hoc* Tukey’s test (*p* < 0.05). Different letters indicate significant differences among regions.

FIGURE S4 Mean relative use for each substrate in each layer. The intensity of color in the boxes indicates the range of values, as indicated in the box. The letters refer to results of a *post hoc* Tukey’s test (*p* < 0.05). Different letters indicate significant differences among depths.

FIGURE S5 Relative use of substrates or mean of categories in the upper 1,000 m smoothed with splines. The green squares mark the range of depths of the deep chlorophyll maximum (19–150 m) and the gray squares mark the range of the deep scattering layer (270–800 m). HU, high-use substrates; LU, low-use substrates.

TABLE S1 Literature using Biolog plates to assess community functional diversity in different marine habitats. Number of natural samples means directly collected from the sea, i.e., in experiments, the initial time.

TABLE S2 Mean values and standard deviation (in brackets) of environmental and biological parameters for the samples collected at each oceanic layer. DCM, Deep Chlorophyll Maximum; Chla, chlorophyll *a* (μg l^–1^); PA: prokaryotic abundance (cell ml^–1^): PHP: prokaryotic heterotrophic production (μg C l^–1^ d^–1^); VA: viral abundance ml^–1^, DOC: dissolved organic carbon (μmol C l^–1^); Fluorescence of DOM components C1, C2, C3, C4 (Raman units). *N* indicates the range in the number of samples analyzed in each layer depending on the variable. Depths of each layer: Surface: 3 m; DCM: 19–150 m; Mesopelagic: 270–980 m; Bathypelagic: 1,000–4,000 m.

TABLE S3 List of substrates in the Biolog GN2 microplates grouped by category.

TABLE S4 Carbon sources included in the two categories for which we divided the carbohydrates and carboxylic acids depending on their mean relative use. HU (high use) are those among the 10 used the most, while LU (low use) are the rest of substrates less used.
