# Supplementary material for: Prokaryotic Capability to Use Organic Substrates Across the Global Tropical and Subtropical Ocean
Source: Front Microbiol. 2020 Jun 4;11:918. doi: 10.3389/fmicb.2020.00918 (PMC7287293; doi:10.3389/fmicb.2020.00918)
Supplement: FIGURE S1 — Relationship between the incubation time of the plates and the number of substrates used (A) and the functional diversity, Shannon (B). [file Data_Sheet_1.DOCX]

**SUPLEMENTARY FIGURES**


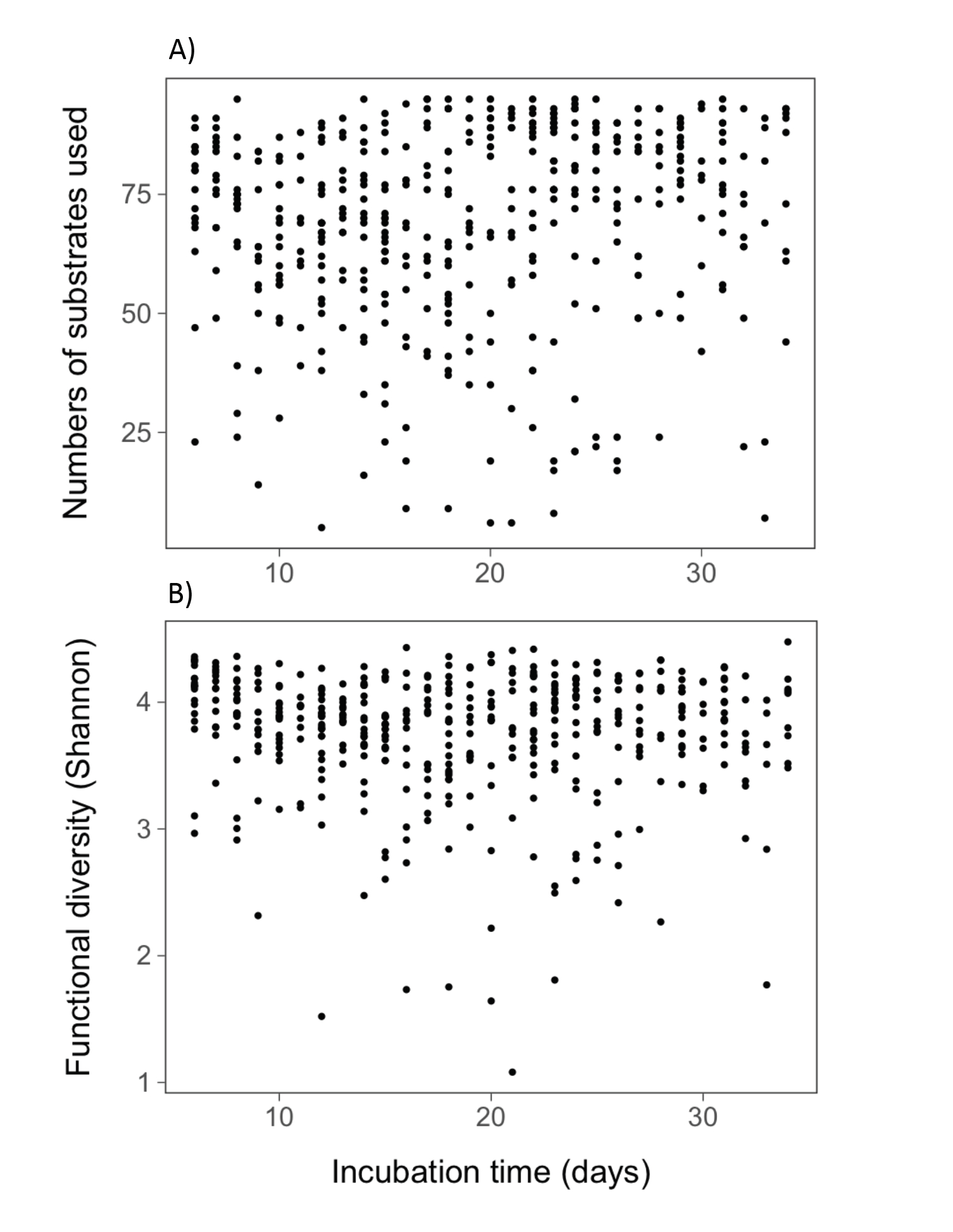


Supp Fig. 1

Supp Fig.2

Mean relative use of substrates

Mean relative use of substrates in Malaspina cruise


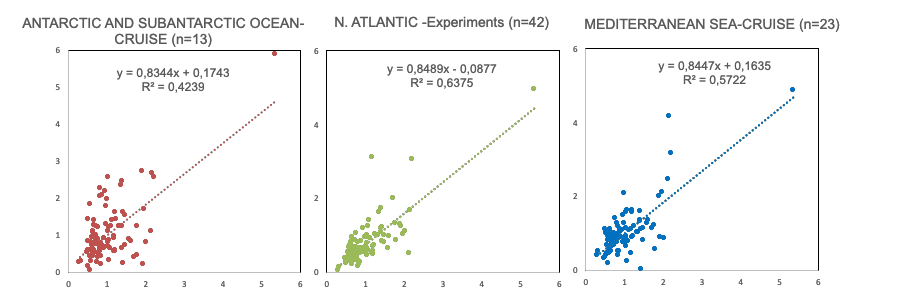


Supp Fig. 2


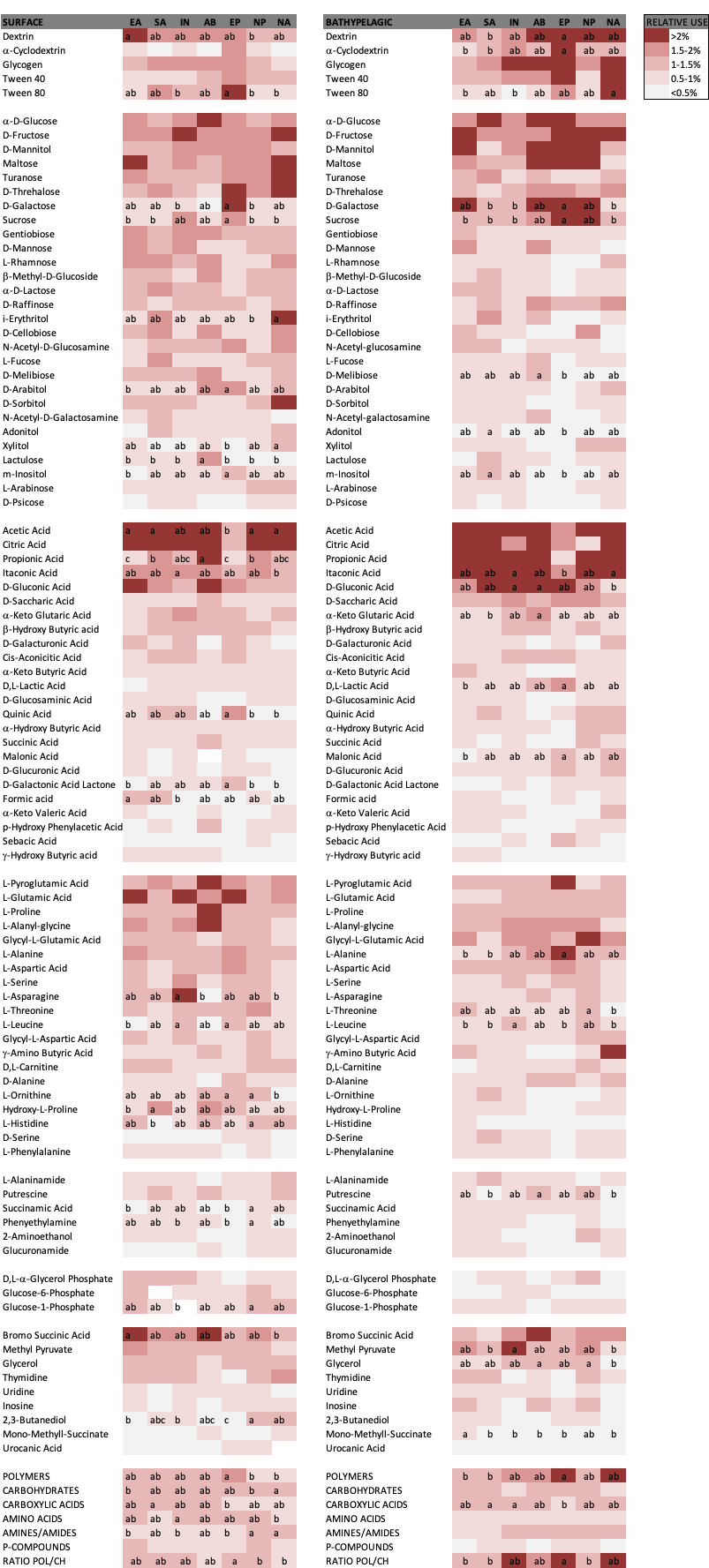


Supp Fig. 3

Fig S4

Supp Fig.7


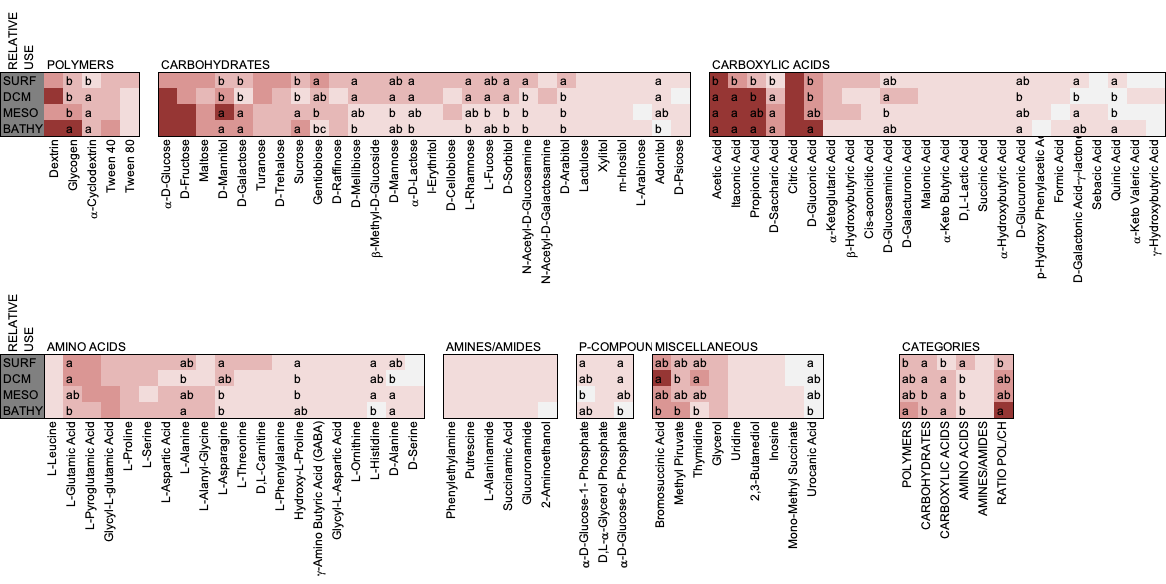


Supp Fig. 4


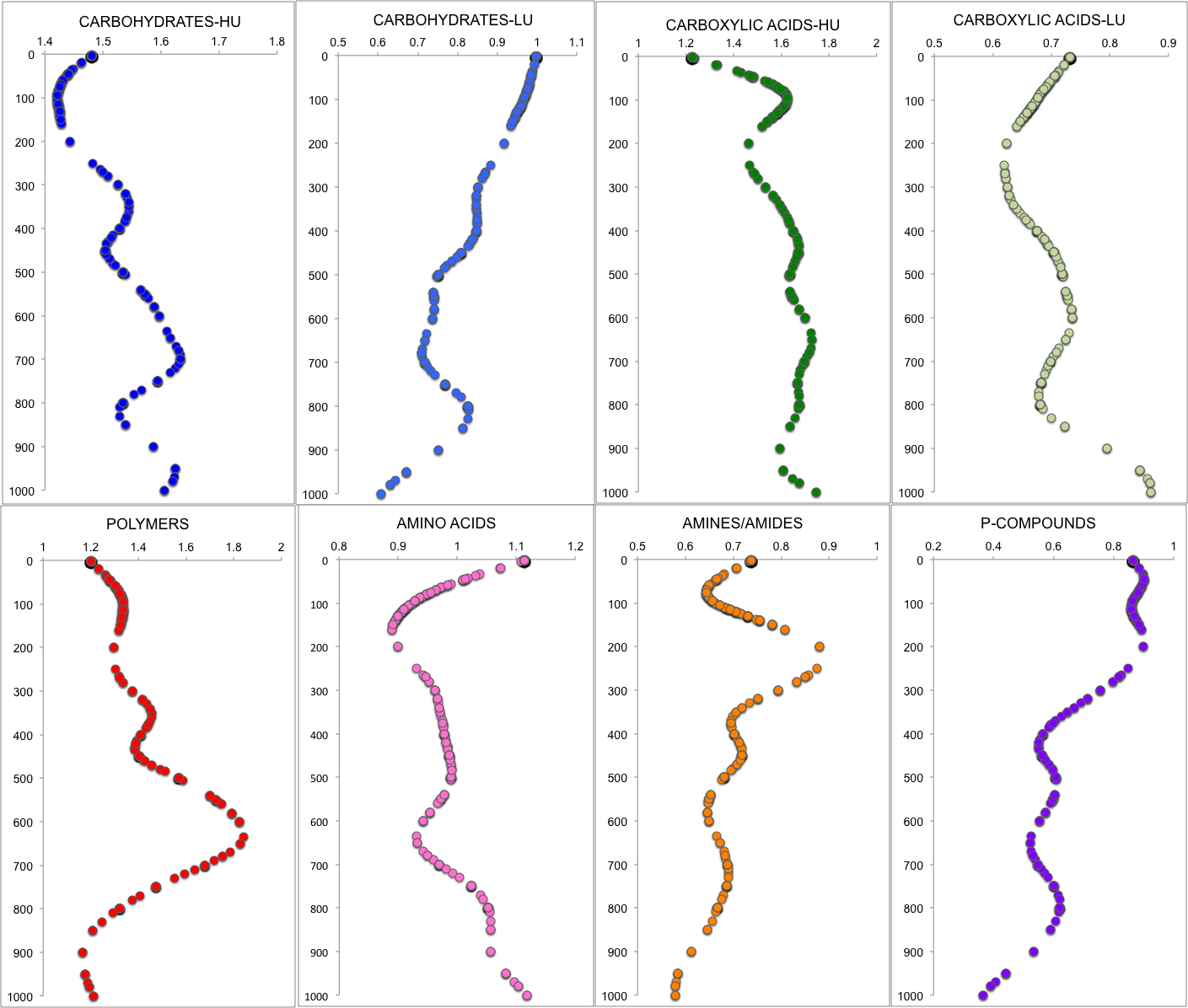


Relative use of substrate or category

Depth

Supp Fig. 5

Supp Fig.5
