# Supplementary material for: Prokaryotic Capability to Use Organic Substrates Across the Global Tropical and Subtropical Ocean
Source: Front Microbiol. 2020 Jun 4;11:918. doi: 10.3389/fmicb.2020.00918 (PMC7287293; doi:10.3389/fmicb.2020.00918)

Supplementary Tables

**Table S1.** Literature using Biolog plates to assess community functional diversity in different marine habitats. Number of natural samples means directly collected from the sea, i.e. in experiments, the initial time.

**Table S2.** Mean values and standard deviation (in brackets) of environmental and biological parameters for the samples collected at each oceanic layer. DCM, Deep Chlorophyll Maximum; Chla, chlorophyll a (μg l^-1^); PA: prokaryotic abundance (cell ml^-1^): PHP: prokaryotic heterotrophic production (μg C l^-1^ d^-1^); VA: viral abundance ml^-1^, DOC: dissolved organic carbon (μmol C l^-1^); Fluorescence of DOM components C1, C2, C3, C4 (Raman units). N indicates the range in the number of samples analyzed in each layer depending on the variable. Depths of each layer: Surface: 3 m; DCM: 19-150m; Mesopelagic: 270-980 m; Bathypelagic: 1000-4000 m.

| LAYER | Chla | Temperature | Salinity | PA | PHP | | VA | | DOC | C1 | C2 | C3 | C4 |
| --- | --- | --- | --- | --- | --- | --- | --- | --- | --- | --- | --- | --- | --- |
| Surface  *n=59-104* | 0.16  (0.12) | 24.96  (2.83) | 35.64  (0.91) | 7.60 x 10^5^  (4.36 x 10^5^) | 2.13  (5.34) | | 8.28 x 10^6^  (7.80 x 10^6^) | | 91.08  (31.98) | 0.004  (0.002) | 0.003  (0.002) | 0.005  (0.003) | 0.012  (0.004) |
| DCM  *n=52-82* | 0.48  (0.21) | 20.98  (3.65) | 35.76  (0.83) | 6.94 x10^5^ (3.80 x10^5^) | 0.61  (0.95) | | 8.53 x 10^6^  (5.09 x 10^6^) | | 82.57  (27.35) | 0,007  (0.003) | 0.007  (0.003) | 0.006  (0.003) | 0.011  (0.003) |
| Mesopelagic  *n=84-134* | 0 | 9.25  (3.06) | 34.80  (0.41) | 1.83 x 10^5^  (1.82 x10^5^) | 0.12  (0.29) | | 1.78 x 10^6^  (2.70 x 10^6^) | | 47.9  (4.80) | 0.010  (0.004) | 0.008  (0.002) | 0.004  (0.002) | 0.005  (0.003) |
| Bathypelagic  *n=95-119* | 0 | 2.61  (1.42) | 34.76  (0.16) | 6.17 x10^4^  (1.03 x10^4^) | 0.01  (0.02) | | 6.28 x 10^5^  (1.37 x 10^5^) | | 42.2  (3.30) | 0.013  (0.002) | 0.009  (0.001) | 0.004  (0.004) | 0.004  (0.003) |
|  |  |  |  |  |  |  | |  | |  |  |  |  |

**Table S3** List of substrates in the Biolog GN2 microplates grouped by category

**Table S4** Carbon sources included in the two categories for which we divided the carbohydrates and carboxylic acids depending on their mean relative use. HU (high use) are those among the 10 used the most, while LU (low use) are the rest of substrates less used.


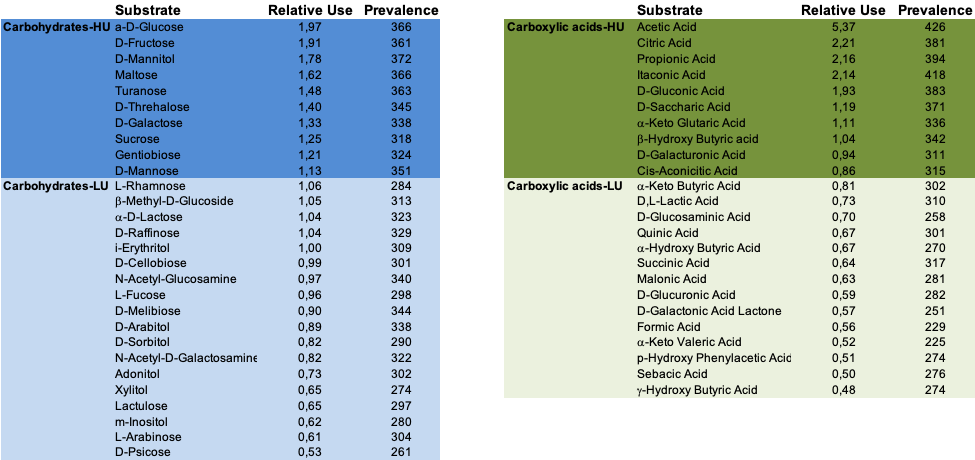

Supplement: TABLE S1 — Literature using Biolog plates to assess community functional diversity in different marine habitats. Number of natural samples means directly collected from the sea, i.e., in experiments, the initial time. [file Data_Sheet_2.DOCX]
