# Supplementary material for: Prokaryotic Capability to Use Organic Substrates Across the Global Tropical and Subtropical Ocean
Source: Front Microbiol. 2020 Jun 4;11:918. doi: 10.3389/fmicb.2020.00918 (PMC7287293; doi:10.3389/fmicb.2020.00918)
Supplement: Supplementary file 3 [file Data_Sheet_3.DOCX]

**SUPPLEMENTARY METHODS (Sala et al.)**

**Temperature and salinity**

Temperature and salinity were measured with the CTD, a SeaBird 9/11-plus.

**Chlorophyll concentration**

To determine total chlorophyll a concentration, a volume of 200-500 ml was filtered through GF/F glass fiber filters and then frozen. Later, the filters were submerged in acetone 90% 24h and left in the fridge 24h. Fluorescence of the samples was measured with a Turner Designs fluorometer, see details in Estrada et al. 2016.

**Prokaryotic and viral abundance**

Prokaryotic and viral abundance (PA and VA) were determined by flow cytometry. Subsamples (2 ml) were fixed with glutaraldehyde for viruses (0.5% final concentration) or paraformaldehyde for bacteria (1% final concentration). Samples of viruses were fixed in the dark at 4ºC for 15–30 min, then quick frozen in liquid nitrogen and stored at -80ºC as in Boras et al. (2010). Samples for bacteria were analysed on board immediately after fixation. Counts for both were made on a FACSCalibur (Becton & Dickinson) flow cytometer. Virus samples were diluted with a TE-buffer (10:1 mM Tris:EDTA), stained with SYBR Green I and run at a medium flow speed (Brussaard, et al. 2004). Prokaryote samples were stained with a DMS (dimethyl sulphate)-diluted SYTO13 and run at a low speed using 0.92-lm yellow-green latex beads as an internal standard (del Giorgio et al. 1996).

**Prokaryotic heterotrophic production.**

Six 1.5 ml vials (4 replicates and 2 killed controls) were filled with 1.2 ml of seawater. A total of 120 μl of cold 50% trichloroacetic acid (TCA) was added to the killed controls. After 15 min, 20 nmol l−1 of L-[4,5-3 H] leucine (144.2 Ci mmol^−1^ , Amersham) was added to all samples, which were incubated for 2.5–6 h at in situ temperature. Incubations were terminated by adding TCA (5% final concentration) to the samples. The prokaryotic cells were pelleted by two successive centrifugation steps (12 000 rpm, 10 min), including a washing step with 1 ml of 5% TCA following Kirchman et al. (1985) with slight modifications (Smith and Azam, 1992). Scintillation cocktail was added to the pellets and radioactivity was measured in a liquid scintillation counter (Wallac-PerkinElmer). For deep samples, 5 ml were incubated and the sample was filtered onto 0.2 μm Nucleopore filters.

**Dissolved organic carbon concentration**

Dissolved organic carbon was collected in pre-combusted 10 mL glass ampoules, acidified to pH < 2 and heat sealed just after collection. Once in the base laboratory, samples were measured by high temperature catalytic oxidation in a Shimadzu TOC-V analyser.

**Fluorescent dissolved organic matter (FDOM)**

The percentage contribution of each component (C1, C2, C3, C4) was calculated relative to the total fluorescence of the four PARAFAC components obtained by Catalá et al. (2015)

**Deep Scattering Layer**

Continuous acoustic measurements were made with a calibrated 42 Simrad EK60 echosounder (7 beam width), operating at a frequency of 38 kHz and with a ping rate of 1 transmitted pulse per 2 s. The data were stored for later analysis, carried out using the LSSS software43. Further details in Asknes et al (2017)
