# Supplementary material for: Prokaryotic Capability to Use Organic Substrates Across the Global Tropical and Subtropical Ocean
Source: Front Microbiol. 2020 Jun 4;11:918. doi: 10.3389/fmicb.2020.00918 (PMC7287293; doi:10.3389/fmicb.2020.00918)
Supplement: Supplementary file 4 [file Data_Sheet_4.DOCX]

**Supplementary literature (Sala et al.)**

References in supplementary figures, tables and material and methods

Aksnes, D. L., Rostad, A., Kaartvedt, S., Martinez, U., Duarte, C. M., & Irigoien, X. (2017). Light penetration structures the deep acoustic scattering layers in the global ocean. Science Advances, 3(5). doi:10.1126/sciadv.1602468

Boras, J. A., Vaqué, D., Maynou, F., Sa, E. L., Weinbauer, M. G., & Sala, M. M. (2015). Factors shaping bacterial phylogenetic and functional diversity in coastal waters of the NW Mediterranean Sea. Estuarine Coastal and Shelf Science, 154, 102-110. doi:10.1016/j.ecss.2014.12.039

Catalá, T. S., Reche, I., Fuentes-Lema, A., Romera-Castillo, C., Nieto-Cid, M., Ortega-Retuerta, E., . . . Älvarez-Salgado, X. A. (2015). Turnover time of fluorescent dissolved organic matter in the dark global ocean. Nature Communications, 6. doi:10.1038/ncomms6986

Estrada, M., Delgado, M., Blasco, D., Latasa, M., Cabello, A.M., Benítez-Barrios, V., . . . Vidal, M. (2016). Phytoplankton across tropical and subtropical regions of the Atlantic, Indian and Pacific oceans. Plos One, 11(3). doi:10.1371/journal.pone.0151699

Fernández-Gómez, B., Sala, M. M., & Pedrós-Alió, C. (2014). Seasonal changes in substrate utilization patterns by bacterioplankton in the Amundsen Gulf (western Arctic). Polar Biology, 37(9), 1321-1329. doi:10.1007/s00300-014-1523-9

Hahn (2003). Alteration of microbial community composition and changes in decomposition associated with an invasive intertidal macrophyte. Biological Invasions 5: 45-51.

Jellett, J. F., Li, W. K. W., Dickie, P. M., Boraie, A., & Kepkay, P. E. (1996). Metabolic activity of bacterioplankton communities assessed by flow cytometry and single carbon substrate utilization. Marine Ecology Progress Series, 136(1-3), 213-225. doi:10.3354/meps136213

Kirchman, D., Knees, E., and Hodson, R. 1985. Leucine incorporation and its potential as a measure of protein-synthesis by bacteria in natural aquatic systems. Applied and Environmental Microbiology, 49: 599–607.

Morris, L; O'Brien, A; Natera, SHA; Lutz, A; Roessner, U; Long, SM (2018). Structural and functional measures of marine microbial communities: An experiment to assess implications for oil spill management 131: 525-529.

Mouchet, M.A.; Bouvier, C.; Bouvier, T.; Troussellier, M.; Escalas, A.; Mouillot, D. (2012). Genetic difference but functional similarity among fish gut bacterial communities through molecular and biochemical fingerprints. FEMS Microbiology Ecology 79: 568-580.

Pineiro-Juncal, N.; Mateo, M.A.; Holmer, M.; Martinez-Cortizas, A. (2018) Potential microbial functional activity along a Posidonia oceanica soil profile. Aquatic Microbial Ecology 81: 189-200.

Richardson, N.F.; Ruesink, J.L.; Naeem, S.; Hacker, S.D.; Tallis, H.M.; Dumbauld, B.R.; Wisehart, L.M. (2008) Bacterial abundance and aerobic microbial activity across natural and oyster aquaculture habitats during summer conditions in a northeastern Pacific estuary. Hydrobiologia 596: 269-278.

Sala, M. M., Arin, L., Balagué, V., Felipe, J., Guadayol, O., & Vaqué, D. (2005). Functional diversity of bacterioplankton assemblages in western Antarctic seawaters during late spring. Marine Ecology-Progress Series, 292.

Sala, M. M., Arrieta, J. M., Boras, J. A., Duarte, C. M., & Vaqué, D. (2010). The impact of ice melting on bacterioplankton in the Arctic Ocean. Polar Biology, 33(12), 1683-1694. doi:10.1007/s00300-010-0808-x

Sala, M. M., Balagué, V., Pedrós-Alió, C., Massana, R., Felipe, J., Arin, L., . . . Estrada, M. (2005). Phylogenetic and functional diversity of bacterioplankton during Alexandrium spp. blooms. Fems Microbiology Ecology, 54(2). doi:10.1016/j.femsec.2005.04.005

Sala, M. M., Estrada, M., & Gasol, J. M. (2006). Seasonal changes in the functional diversity of bacterioplankton in contrasting coastal environments of the NW Mediterranean. Aquatic Microbial Ecology, 44(1), 1-9. doi:10.3354/ame044001

Sala, M. M., Terrado, R., Lovejoy, C., Unrein, F., & Pedrós-Alió, C. (2008). Metabolic diversity of heterotrophic bacterioplankton over winter and spring in the coastal Arctic Ocean. Environmental Microbiology, 10(4), 942-949. doi:10.1111/j.1462-2920.2007.01513.x

Sawstrom, C.; Serrano, O.; Rozaimi, M.; Lavery, P.S. (2016). Utilization of carbon substrates by heterotrophic bacteria through vertical sediment profiles in coastal and estuarine seagrass meadows. Environmental Microbiology Reports 8: 582-589

Smith, D. C., and Azam, F. 1992. A simple, economical method for measuring bacterial protein synthesis rates in seawater using 3H-leucine. Marine Microbial Food Webs, 6: 107–114.

Tam, L., Kevan. P.G., Trevors, J.T. (2003). Viable bacterial biomass and functional diversity in fresh and marine waters in the Canadian Arctic. Polar Biology, 26: 287-294.

They, N.H., Ferreira, L.M.H., Marins, L.F., Abreu, P.C. (2013) Stability of bacterial composition and activity in different salinity waters in the dynamic Patos Lagoon estuary: evidence from a lagrangian-like approach. Microbial Ecology, 66: 551-562.

Wear, E. K., Koepfler, E. T., & Smith, E. M. (2014). Spatiotemporal variability in dissolved organic matter composition is more strongly related to bacterioplankton community composition than to metabolic capability in a blackwater estuarine system. Estuaries and Coasts, 37(1), 119-133. doi:10.1007/s12237-013-9651-y

Wurl. O., Stolle, C., Van Thuoc, X., The Thu, P., Mari, X. (2016). Biofilm-like properties of the sea surface and predicted effects on air–sea CO_2_ exchange. Progress in Oceanography, 144: 15-24.

Yang, Y.F; Hu, X.J.; Zhang, J.; Gong, Y.X. (2013) Community level physiological study of algicidal bacteria in the phycospheres of Skeletonema costatum and Scrippsiella trochoidea. Harmful Algae 28: 88-96.
